# Microencapsulation of *Lacticaseibacillus rhamnosus* GG for Oral Delivery of Bovine Lactoferrin: Study of Encapsulation Stability, Cell Viability, and Drug Release

**DOI:** 10.3390/biomimetics7040152

**Published:** 2022-10-04

**Authors:** Yasir Anwar, Ihsan Ullah, Tahseen Kamal, Muhammad Wajid Ullah

**Affiliations:** 1 Department of Biological Sciences, Faculty of Science, King Abdulaziz University, Jeddah 21589, Saudi Arabia; 2 Center of Excellence for Advanced Materials Research, King Abdulaziz University, Jeddah 21589, Saudi Arabia; 3 Biofuels Institute, School of the Environment and Safety Engineering, Jiangsu University, Zhenjiang 212013, China

**Keywords:** probiotics, microencapsulation, gelation, crosslinking, mineralization, swelling, cell viability, drug delivery

## Abstract

Probiotics are delivered orally for treating gastrointestinal tract (GIT) infections; thus, they should be protected from the harsh environment of the GIT, such as through microencapsulation. Here, we microencapsulated cells of the probiotic *Lacticaseibacillus rhamnosus* GG via the liquid-droplet-forming method and evaluated them for oral delivery of bovine lactoferrin (bLf). Briefly, sodium alginate capsules (G-capsules) were first prepared, crosslinked with calcium chloride (C-capsules), and then modified with disodium hydrogen phosphate (M-capsules). All capsules showed good swelling behavior in the order of G-capsules > C-capsules > M-capsules in simulated gastric fluid (SGF, pH 2) and simulated intestinal fluid (SIF, pH 7.2). FE-SEM observations showed the formation of porous surfaces and successful microencapsulation of *L. rhamnosus* GG cells. The microencapsulated probiotics showed 85% and 77% viability in SGF and SIF, respectively, after 300 min. Compared to the 65% and 70% viability of gelation-encapsulated and crosslinking-encapsulated *L. rhamnosus* GG cells, respectively, the mineralization-encapsulated cells showed up to 85% viability after 300 min in SIF. The entrapment of bLf in the mineralization-encapsulated *L. rhamnosus* GG cells did not show any toxicity to the cells. FTIR spectroscopy confirmed the successful surface modification of *L. rhamnosus* GG cells via gelation, crosslinking, and mineralization, along with the entrapment of bLf on the surface of microencapsulated cells. The findings of these studies show that the microencapsulated *L. rhamnosus* GG cells with natural polyelectrolytes could be used as stable carriers for the oral and sustainable delivery of beneficial biotherapeutics without compromising their viability and the activity of probiotics.

## 1. Introduction

Probiotics refer to food or supplements containing live microorganisms that maintain the human microbiota. They are orally delivered to treat gastrointestinal tract (GIT) infections by minimizing pathogenic bacteria [1]. Moreover, they are used as food additives because of their ability to serve as a barrier against pathogen invasion, alleviate antibiotic-associated diarrhea, and stimulate immunomodulation [2]. Although oral delivery is a simple, convenient, and low-cost approach with a minimal risk of infection [3], the orally delivered protein-based drugs suffer from a low level of bioavailability, poor absorption, internalization through the GIT epithelium, and high hydrolytic and enzymatic [4] degradation by the GIT fluids [5,6]. At the same time, the oral administration of probiotics is a major challenge considering the risks of systemic infection, replacement of the human microbiota, gene transfer, deleterious metabolic activities, low survival in the GIT, provoking the immune response, etc. [7,8,9]. Similarly, the activity and viability of most probiotic bacteria are affected by severe GIT conditions such as oxygen, acid, heat, bile salt, and pressure during their oral administration [10] and should thus be protected, e.g., through surface modification or microencapsulation [11].

Among the different techniques for cell surface modification, including layer-by-layer [12], extrusion [13], genetic engineering [14], adsorption [15], biomineralization [16], direct single-step magnetization [4], etc., microencapsulation has been widely employed in the food industry for enhancing the survival rates of probiotics [17]. Microencapsulation of probiotics not only provides protection to the bacterial cells, but also assists the probiotic-specific delivery [18]. The encapsulating layer serves as a protective barrier between the probiotics and the environment, enhancing their survival under the harsh conditions of the GIT following their oral delivery. This layer also hinders the direct contact of probiotics with the intestinal lining. Microencapsulation allows a controlled release of the encapsulated material under the influence of environmental conditions such as pH, temperature, ionic strength, electrical stimuli, etc. [19]. This controlled release requires that the microencapsulating materials are semi-permeable, with a diameter of a few micrometers, and form a strong membrane surrounding a liquid/solid core [20]. A variety of materials of different natures—such as polyelectrolytes, colloids, and macromolecules—have been used for the development of biocompatible and semi-permeable microencapsulation systems for the encapsulation of viable bacteria [21,22]. These materials form artificial cells, capsules, beads, etc., according to the fabrication strategy. Among the different polymers, alginate has been widely used in microencapsulation and other fabrication processes due to its biocompatible nature and gel-forming ability [23,24,25]. Alginate forms a strong hydrogel with calcium chloride ions in the dispending-based fabrication process. The calcium ions can further crosslink with phosphate and form hybrid nanoparticles through self-assembly [26,27]. Microencapsulation not only protects the probiotics from harsh environmental conditions such as enzymatic and chemical reactions, pH and temperature variation, heat, and mechanical pressure—as encountered in the GIT [28]—but also controls the movement of ions and solutes to regulate their metabolism. Such microencapsulation systems could be used for the oral delivery of protein-based drugs. To date, alginate-based matrices and nanoparticles have been used for the oral delivery of vaccines, polynucleotides, and proteins [29,30], owing to their biocompatible nature and capsule-forming ability.

Although polymer-encapsulated probiotics have known roles in treating various diseases, their use as carriers for the delivery of other beneficial biotherapeutics has not been reported. *L. rhamnosus*, reclassified as *Lacticaseibacillus rhamnosus* [31], is commonly found in the mouth and considered a ‘good’ bacterium that breaks down the food contents, absorbs nutrients, and also fights the ‘bad’ bacteria. It is added to fermented foods such as yogurt and is commonly used in probiotic supplements [32]. Therefore, the present study aimed to microencapsulate *L. rhamnosus* GG with a single layer of polyelectrolytes, including sodium alginate, calcium chloride, and disodium hydrogen phosphate, to establish a system for the simultaneous oral delivery of probiotics (i.e., *L. rhamnosus* GG) and drugs (i.e., bovine lactoferrin (bLf)). Three types of capsules were prepared: First, G-capsules were prepared through the liquid-droplet-forming method, which were then modified into C-capsules through crosslinking with calcium chloride. Finally, M-capsules were obtained through mineralization of C-capsules. The surface morphologies of all types of capsules were observed via FE-SEM. All types of capsules were evaluated for swelling behavior in simulated intestinal fluid (SIF, pH 7.2) and simulated gastric fluid (SGF, pH 2) and used for the encapsulation of *L. rhamnosus* GG cells. The morphologies of the encapsulated *L. rhamnosus* GG were observed through FE-SEM, while the chemical interaction between the encapsulating materials themselves and the cells was determined through Fourier-transform infrared (FTIR) spectroscopy. The encapsulated cells were evaluated for their bLf entrapment efficiency and release profile. We further studied the effects of encapsulation, encapsulating materials, and the toxicity of the entrapped bLf on the viability of encapsulated *L. rhamnosus* GG. The findings of this study show that encapsulation of *L. rhamnosus* GG with polyelectrolytes did not affect the cell viability and showed a controlled release of bLf; thus, this technique could be used for the oral delivery of other biotherapeutics.

## 2. Materials and Methods

### 2.1. Materials

Sodium alginate (C_6_H_7_NaO_6_)_n_ (molecular weight 196 kDa), calcium chloride (CaCl_2_), and disodium hydrogen phosphate dodecahydrate (Na_2_HPO_4_.12H_2_O) were purchased from Sinopharm Chemical Reagent Co., Ltd. (Shanghai, China). Xanthan gum was provided by Lavendersoap (New York, NY, USA). Tween 20 was purchased from Duksan Pharmaceutical Co., Ltd. (Ansan-si, South Korea). Bovine lactoferrin (bLf) was supplied by Sigma-Aldrich (St. Louis, MO, USA). Agar and de Man–Rogosa–Sharpe (MRS) broth were supplied by Solar-Bio Science and Technology Co., Ltd. (Beijing, China). All other reagents and chemicals used were of analytical grade and used without further processing unless otherwise stated.

### 2.2. Preparation of SGF and SIF

The SGF and SIF solutions were prepared according to a previously reported method [33]. Briefly, the simulated gastric fluid (SGF) was prepared by dissolving 1 wt.% porcine pepsin in diluted hydrochloric acid (pH 2). Similarly, the simulated intestinal fluid (SIF) was prepared by dissolving 1 wt.% porcine pancreatin in a solution containing 6.804 g L^−1^ KH_2_PO_4_ (pH 7.2). Finally, the desired pH of each solution was adjusted by using 0.1 M HCl and 0.2 M NaOH.

### 2.3. Preparation and Characterization of Capsules

**Figure 1:**
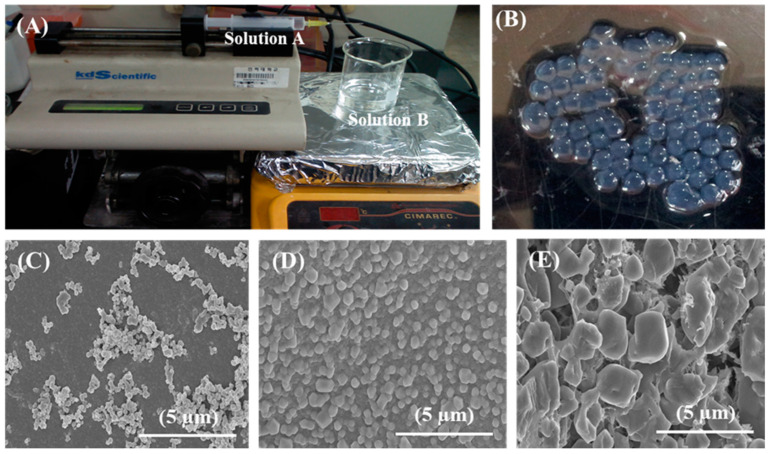
Preparation and structural features of microcapsules: Digital images of (**A**) assembly of preparation and (**B**) prepared G-capsules via the liquid-droplet-forming method. FE-SEM micrographs of the surface morphologies of (**C**) G-capsules, (**D**) C-capsules, and (**E**) M-capsules.

First, sodium alginate capsules were prepared via the liquid-droplet-forming method, as reported in our previous study [24]. First, two solutions—solution A and solution B—were prepared. Solution A was composed of 1 wt.% CaCl_2_ and 0.15 wt.% xanthan gum, while solution B contained 0.6 wt.% sodium alginate and 0.1% Tween 20. For the preparation of the capsules, 4 mL of solution A was loaded in a 22-gauge blunt-ended needle and added dropwise to 30 mL of solution B under stirring at 700 rpm using a 2 cm long magnetic bar, according to the assembly shown in Figure 1A. The capsules were immediately formed and named G-capsules (‘G’ denotes ‘gelation’). The capsules were then moved to a 2 wt.% CaCl_2_ solution and allowed to crosslink for 10 min. The excess sodium alginate was removed by washing the capsules with sterile saline. The C-capsules were again resuspended in 2 wt.% CaCl_2_ under stirring at 700 rpm for 15 min to allow them to harden and then washed with saline after harvesting. These capsules were named C-capsules (‘C’ denotes ‘crosslinking’). Finally, the capsules were mineralized by adding 0.5 wt.% Na_2_HPO_4_ under stirring at 700 rpm for 10 min and then allowed to settle down. Finally, the capsules were harvested and washed three times with saline to remove the excess Na_2_HPO_4_. These capsules were named M-capsules (‘M’ denotes ‘mineralization’).

The surface morphologies of all types of freeze-dried capsules were observed under FE-SEM (Hitachi S-4800, Hitachi, Tokyo, Japan). For FE-SEM observation, the capsules were fixed onto a brass holder and coated with gold before observation.

### 2.4. Swelling Behavior of Capsules

The swelling behavior of G-capsules, C-capsules, and M-capsules was separately determined in SIF (pH 7.2) and SGF (pH 2). Briefly, the capsules were separately added to tubes containing 25 mL of SIF or SGF and incubated at 37 °C under shaking at 700 rpm. The weight of the capsules in the SGF and SIF solutions was determined at different time intervals. The swelling (%) was determined as the weight difference at the time ‘0’ (i.e., W_0_) and time ‘t’ (W_t_) by using Equation (1):

(1) $$\mathrm{Swelling}\;\left(\%\right)=\frac{W_{t}}{W_{o}}\times 100$$

### 2.5. Culturing and Microencapsulation of L. rhamnosus GG

The *L. rhamnosus* GG culture was obtained from the China Center of Industrial Culture Collection and grown in 60 g/L sterilized (121 °C for 15 min) RMS broth. Briefly, 3 mL of *L. rhamnosus* GG culture was inoculated into the MRS broth and incubated statically overnight at 30 °C. After incubation, the broth was centrifuged at 9000 rpm for 20 min. The obtained pellet was washed three times with saline. Thereafter, the cells were encapsulated by following the procedures described for the preparation of G-capsules, C-capsules, and M-capsules (Section 2.2), except that the physical mixing of *L. rhamnosus* GG with different components of the capsules was carried out without employing the liquid-droplet-forming method.

### 2.6. Entrapment of bLf on the Surface of Encapsulated L. rhamnosus GG, and Chemical Characterization

For the entrapment of bLf on the surface of encapsulated *L. rhamnosus* GG, the cells encapsulated in G-capsules, C-capsules, and M-capsules were separately mixed with 10 mL of 2 mg/mL bLf solution prepared in phosphate-buffered saline (PBS) (pH 7.4). The encapsulated *L. rhamnosus* GG cells were incubated under shaking at 150 rpm for 12 h at 37 °C. After incubation, the encapsulated cells with entrapped bLf were harvested via centrifugation at 9000 rpm for 20 min. The entrapment efficiency of bLf in the surface-modified *L. rhamnosus* GG cells was evaluated by determining the bLf concentration in the supernatant after harvesting of the cells via the Bradford assay [34]. Briefly, the bLf-entrapped encapsulated *L. rhamnosus* GG cells were harvested via centrifugation at 9000 rpm for 20 min, and the supernatant was decanted. For analysis, 20 µL of supernatant was placed in a 96-well plate, and 200 µL of Bradford reagent was added to the well. The mixture was thoroughly mixed for 20 s and incubated at 25 °C for 5 min. The absorbance was measured at 595 nm by using a UV–visible spectrophotometer. The protein standards prepared in 0–2 µL of buffer were used for reference. The entrapment efficiency of bLf in surface-modified encapsulated *L. rhamnosus* GG was determined as the difference between the total bLf added to the system and the amount present in the supernatant after incubation by using Equation (2):

(2) $$\mathrm{Entrapment}\;\mathrm{efficiency}\;\left(\%\right)=\frac{\mathrm{Total}\;\mathrm{bLf}-\mathrm{Resiidual}\;\mathrm{bLf}\;\mathrm{in}\;\mathrm{supernatant}}{\mathrm{Total}\;\mathrm{bLf}\;}\times 100$$

The entrapment of bLf and its possible interaction with the components of the capsules on the surface of encapsulated *L. rhamnosus* GG in G-capsules, C-capsules, and M-capsules was confirmed through FTIR spectroscopy by using a PerkinElmer FTIR infrared spectrometer (VERTEX 70, Germany) in the region of 400–4000 cm^−1^ (beam splitter: Ge coated on KBr; detector: DTGS; resolution: 0.25 cm^−1^ (step selectable)). For analysis, both the bLf-entrapped and non-bLf-entrapped encapsulated *L. rhamnosus* GG cells were isolated via centrifugation at 9000 rpm for 20 min and then freeze-dried for 24 h.

### 2.7. In Vitro bLf Release

For in vitro release analysis of bLf, the bLf-entrapped encapsulated *L. rhamnosus* GG cells were added to 10 mL of sterile SGF and incubated at 37 °C for 2 h, followed by incubation in SIF for 22 h under shaking at 150 rpm. At predetermined time points, a 1 mL aliquot of the sample was withdrawn, and the mixture was replenished with the same amount of PBS to maintain the total volume. The concentration of bLf in the aliquot was determined via the Bradford assay, as described in Section 2.5.

### 2.8. Viability of L. rhamnosus GG after the Release of bLf

The viability of encapsulated *L. rhamnosus* GG cells after 24 h of the release of bLf was determined via the agar plate culturing method. The M-capsule-entrapped cells were harvested via centrifugation at 9000 rpm for 20 min and washed three times with sterile water. The obtained cell pellet was serially diluted in normal saline and spread on an MRS agar plate. The agar plate was then incubated overnight at 37 °C. The cell viability was determined quantitatively every 60 min for 300 min. The effect of different encapsulation materials on cell viability was also determined, as reported previously [35]. Herein, the non-encapsulated *L. rhamnosus* GG cultured in MRS broth was used for reference.

### 2.9. Statistical Analysis

All experiments were performed in triplicate, and the results are shown as the mean values and standard deviations of the results in the reference and treatment groups.

## 3. Results

### 3.1. Formation, Structural Morphology, and Swelling Behavior of Capsules

The formation of alginate microcapsules is a well-established mechanism, as described in our previous report [24]. During the formation of alginate capsules (3.20 ± 0.22 mm), a capsular membrane was immediately formed upon the dropwise addition of CaCl_2_ to the sodium alginate solution, resulting in the formation of G-capsules (Figure 1B). The thickness of the membrane increased until the Ca^2+^ ions within the core of the capsules were exhausted and formed C-capsules of 3.25 ± 0.31 mm in diameter. Finally, the precipitation of Na_2_HPO_4_ on the C-capsules formed a mineralized layer and formed M-capsules of 3.38 ± 0.35 mm in diameter. These results show that the size of the C-capsules only slightly increased upon crosslinking. On the other hand, the precipitation of Na_2_HPO_4_ on the C-capsules greatly increased the capsules’ size, which could be attributed to the formation of an additional mineralization layer on the M-capsules. The size distribution of different capsules formed in this study is summarized in Table 1. The surface morphology of G-capsules observed through FE-SEM showed variably distributed polyhedral particles with a slightly porous surface (Figure 1C), which could be attributed to the strong crosslinking between the components of the capsules, i.e., metal ions (i.e., Ca) and the polymer molecules (i.e., alginate). In contrast, G-capsules showed a more homogenous arrangement and compact structure comprising polyhedral particles (Figure 1D). The FE-SEM micrograph of the M-capsules shows the formation of a highly porous surface with rough shells of variable shapes and sizes (Figure 1E).

**Figure 2:**
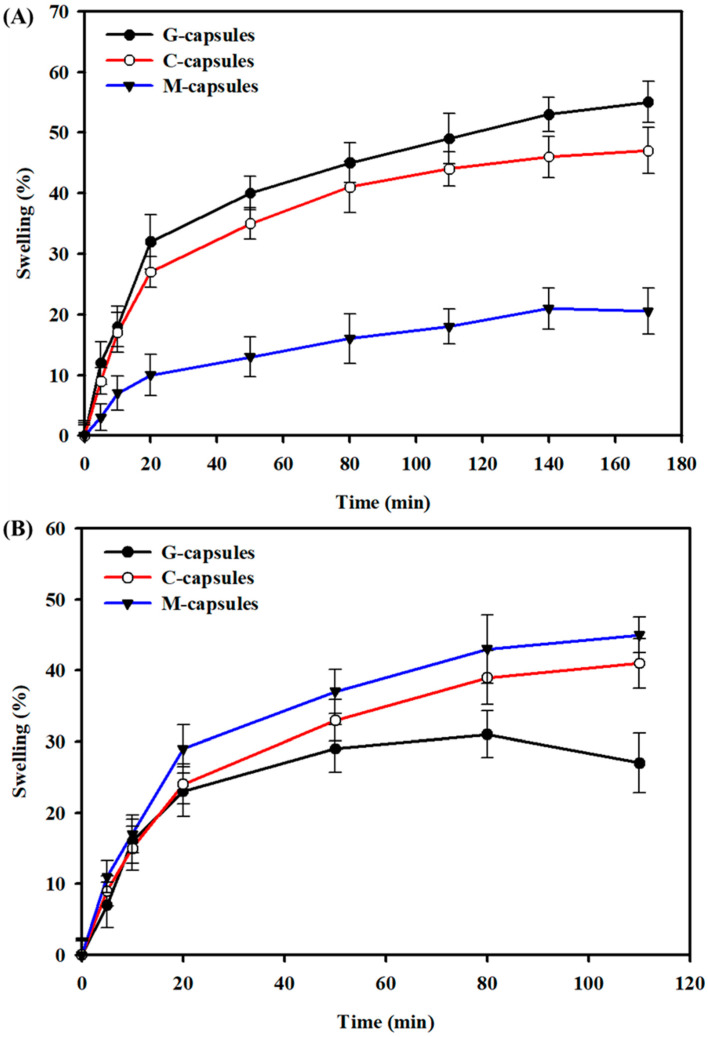
Swelling behavior of G-capsules, C-capsules, and M-capsules: The swelling behavior of capsules in (**A**) simulated gastric fluid (pH 2) and (**B**) simulated intestinal fluid (pH 7.2) as a function of time.

The swelling behavior of G-capsules, C-capsules, and M-capsules was determined in SIF (pH 7.2) and SGF (pH 2) to mimic the pH levels within the GIT, and the results are shown in Figure 2. The results show that G-capsules and C-capsules showed high swelling ability in SGF, with G-capsules > C-capsules, indicating that these can remain stable in the stomach environment. This high swelling ability and stability of both capsule types could be attributed to the acidic gel character of alginate below the pKa value of uronic acid residues (~3.5). There is a direct association between the molecular structure of alginate and its gelation characteristics in acidic conditions. Alginate solution forms a gel at a lower pH, below the pKa value of uronic acid residues [36,37]. The M-capsules showed relatively low swelling behavior compared to both the G-capsules and C-capsules in SGF. This could be due to the acidic behavior of Na_2_HPO_4_ salt, which increases the overall pH of the medium and may also interfere with the swelling ability of the capsules; this may lead to mechanical damage or breakage of the capsules [38]. On the other hand, G-capsules showed a relatively low swelling behavior compared to the C-capsules and M-capsules, with C-capsules < M-capsules, and were dissolved much earlier when immersed in SIF.

### 3.2. Morphology of Encapsulated L. rhamnosus GG Cells

**Figure 3:**
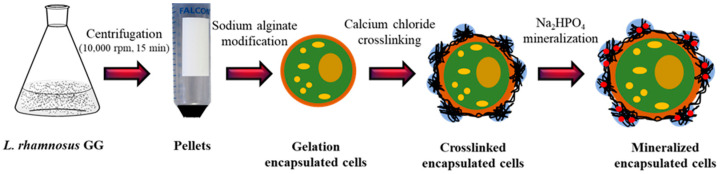
Schematic illustration of the encapsulation of *L. rhamnosus* GG: The *L. rhamnosus* GG cells obtained through centrifugation of broth were modified with a single layer of polyanionic sodium alginate (i.e., gelation), followed by the formation of a polycation layer by adding calcium chloride (i.e., crosslinking) and, finally, forming a layer of disodium hydrogen phosphate (i.e., mineralization).

*L. rhamnosus* GG is a rod-shaped bacterium that varies from 0.8 to 1.0 μm in width and 2 to 4 μm in length. The *L. rhamnosus* GG cells obtained via centrifugation were successfully encapsulated via a three-step process—gelation, crosslinking, and mineralization—as illustrated in Figure 3. The pellet composed of bacterial cells was resuspended in CaCl_2_ solution, and the capsules were formed via the liquid-droplet-forming method. The stability of the capsules containing bacterial cells was further enhanced through additional crosslinking with CaCl_2_ and mineralization with Na_2_HP_4_. The detailed mechanisms of gelation, crosslinking, and mineralization are described in the Discussion section.

**Figure 4:**
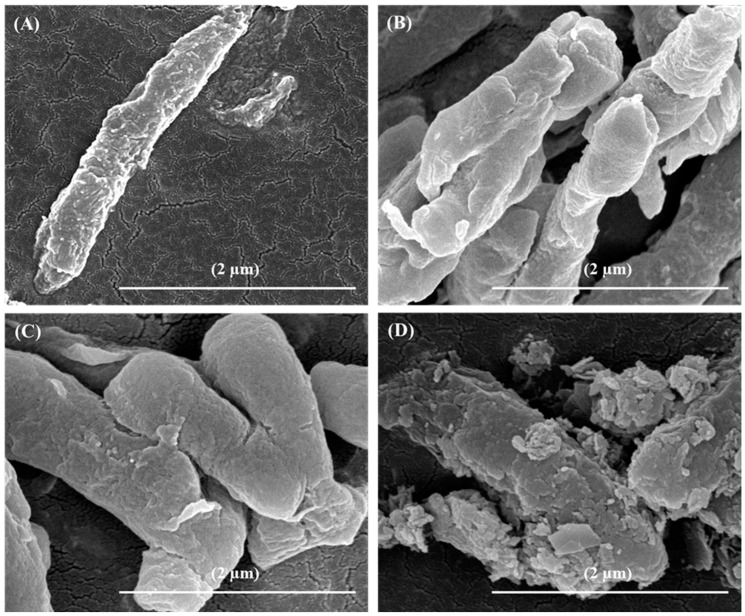
FE-SEM observation of the surface morphologies: The surface morphologies of the (**A**) non-encapsulated, (**B**) gelation-encapsulated, (**C**) crosslinking-encapsulated, and (**D**) mineralization-encapsulated *L. rhamnosus* GG cells.

The structural morphologies of the non-encapsulated and encapsulated *L. rhamnosus* GG observed under FE-SEM are shown in Figure 4. FE-SEM observation of non-encapsulated *L. rhamnosus* GG cells showed a typical rod-shaped bacterium (Figure 4A). Figure 4B shows the gelation-encapsulated *L. rhamnosus* GG cells with a clear layer of calcium alginate, indicating the successful gelation of cells. Although there was no visible difference in morphology between the gelation-encapsulated and crosslinking-encapsulated *L. rhamnosus* GG cells (Figure 4C), the latter showed a more porous and compact surface appearance, in accordance with the FE-SEM observations of the G-capsules and C-capsules, respectively (Figure 1D). The FE-SEM micrograph of mineralization-encapsulated *L. rhamnosus* GG showed the appearance of a mineralized structure, indicating the successful mineralization of cells (Figure 4D). This observation is in accordance with the FE-SEM micrograph of M-capsules (Figure 1E). These results are consistent with previous reports where different cells were successfully encapsulated in different polyelectrolytes and polymers that showed cellular morphologies and functionalities [12,39,40].

### 3.3. Viability of Microencapsulated L. rhamnosus GG Cells

**Figure 5:**
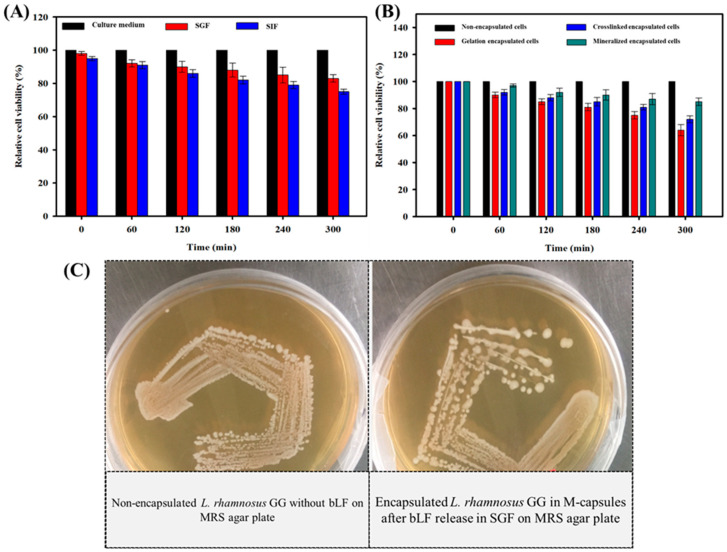
Viability analysis of encapsulated *L. rhamnosus* GG cells: (**A**) Effects of encapsulation on cell viability in SGF and SIF. The bare *L. rhamnosus* GG cells were grown in MRS broth for reference. (**B**) Effects of modifying materials on the viability of cells. The growth of gelation-encapsulated, crosslinking-encapsulated, and mineralization-encapsulated *L. rhamnosus* GG cells was determined in SGF. The bare *L. rhamnosus* GG cells were grown in MRS broth for reference. (**C**) Effect of the toxicity of bLf on the growth of bLf-entrapped mineralization-encapsulated *L. rhamnosus* GG cells. The growth of *L. rhamnosus* GG cells encapsulated in bLf-entrapped M-capsules in SGF on an MRS agar plate after the release of bLf.

The viability of encapsulated *L. rhamnosus* GG cells was determined in SGF (pH 2) and SIF (pH 7.2). The results showed that the encapsulated *L. rhamnosus* GG cells largely remained viable for an extended time period. The cells showed 85% and 77% viability of *L. rhamnosus* GG in SGF and SIF, respectively, after 300 min (Figure 5A). Likewise, the viability of *L. rhamnosus* GG cells encapsulated in a layer composed of alginate, calcium chloride, and disodium hydrogen phosphate was determined in SGF. Herein, SGF was chosen over SIF because of the relatively high viability of *L. rhamnosus* GG cells. The results showed that compared to the gelation-modified (65%) and crosslinking-modified (70%) *L. rhamnosus* GG cells, the mineralization-modified cells showed up to 85% viability after 300 min in SGF (Figure 5B). The growth of bLf-entrapped mineralization-encapsulated *L. rhamnosus* GG cells was also determined after the release of bLf. In Figure 5C, the formation of bacterial colonies on the MRS agar plate shows their viability, indicating the nontoxic effect of bLf on the growth of encapsulated *L. rhamnosus* GG cells.

### 3.4. Entrapment Efficiency and In Vitro Release of bLf from the Encapsulated L. rhamnosus GG Cells

The drug loading and release efficiency of the encapsulating layer composed of sodium alginate, calcium chloride, and disodium hydrogen phosphate were evaluated for bLf. The results for the amount of bLf entrapped on the surface of encapsulated *L. rhamnosus* GG cells are provided in Table 1, which shows that the gelation-encapsulated, crosslinking-encapsulated, and mineralization-encapsulated *L. rhamnosus* GG cells entrapped 78 ± 2.25%, 88 ± 1.78%, and 95 ± 1.12%, respectively, of the initially added bLf to the respective cell suspensions. The entrapment of bLf in the encapsulating layer increased the capsules’ size. The size of the gelation-encapsulated cells, crosslinking-encapsulated cells, and mineralization-encapsulated cells was increased from 3.20 ± 0.22 mm, 3.25 ± 0.31, and 3.38 ± 0.35, respectively, to 3.41 ± 0.22 mm, 3.43 ± 0.18 mm, and 3.61 ± 0.45 mm, respectively. The increase in capsule size could be attributed to the swelling of the capsules during the drug loading.

**Figure 6:**
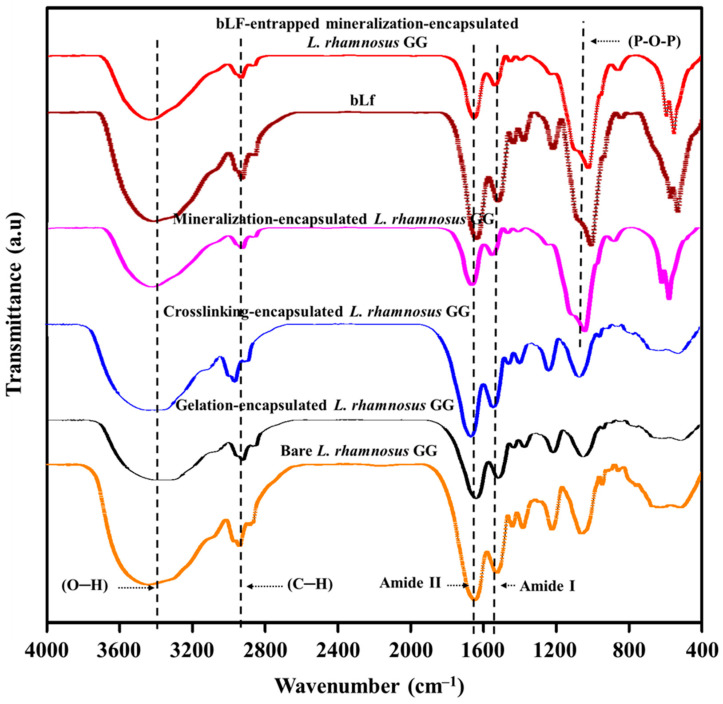
FTIR spectroscopic analysis of the interactions between the surface of *L. rhamnosus* GG cells, modifying materials, and drugs: Chemical structural analyses of bare *L. rhamnosus* GG cells, gelation-encapsulated *L. rhamnosus* GG cells, crosslinking-encapsulated *L. rhamnosus* GG cells, mineralization-encapsulated *L. rhamnosus* GG cells, bLf, and bLf-entrapped mineralization-encapsulated *L. rhamnosus* GG cells.

The entrapment of bLf on the surface of encapsulated *L. rhamnosus* GG cells and the possible chemical interactions between the drug molecules and the chemical functional groups on the modified cell surface were determined by FTIR, and the results are shown in Figure 6. The FTIR spectrum of bare *L. rhamnosus* GG cells showed characteristic absorption bands for the hydroxyl (O–H) group at 3394 cm^−1^. The typical peaks for amide I and amide II functional groups at 1510 cm^−1^ and 1992 cm^−1^, respectively, appeared in the FTIR spectrum of *L. rhamnosus* GG cells due to the presence of proteins in the cell membrane. The gelation-encapsulated *L. rhamnosus* GG cells showed a decreased intensity and a slight shift of the O–H peaks to a lower wavelength, indicating a chemical interaction of sodium ions with the functional groups on the cell membrane and formation of a gelation surface [41]. However, the transformation of gelation-encapsulated cells to crosslinking-encapsulated cells resulted in increased intensity and a slight shift of the O–H group to a higher wavelength, indicating the formation of a chelating structure between the hydroxyl and carboxylate functional groups of alginate and Ca^2+^ ions. This chemical interaction further strengthens the hydrogen bonding on the cell surface [12,42,43]. The mineralization of cells was chemically verified by the presence of specific peaks at 1033 cm^−1^ and 1030 cm^−1^ for phosphate group single bonds, which could be ascribed to the asymmetric stretching vibration of the P–O–P functional group and symmetric stretching of the non-bridging oxygen in the Q^0^ tetrahedron [44]. Moreover, the gelation-encapsulated, crosslinking-encapsulated, and mineralization-encapsulated *L. rhamnosus* GG cells showed peaks at 2927 cm^−1^, 2928 cm^−1^, and 2925 cm^−1^, respectively, for the CH_2_ group of sodium alginate, while the peaks for the amide Iα helical protein structure for the carbonyl (C=O) group appeared at 1655 cm^−1^ in the differently modified cells.

The FTIR spectrum of free bLf showed the characteristic protein absorption bands [45], including the peak for amide I at 1634 cm^−1^ due to the carbonyl stretching vibration of the peptide group and the peak for amide II at 1512 cm^−1^ due to the N–H bending and C–N stretching vibrations. In addition, a strong band for O–H stretching in water molecules appeared at 3332 cm^−1^, indicating the presence of residual water. Furthermore, the bands for asymmetric and symmetric stretching vibration of CH_2_ groups of alkyl chains appeared at 2937 cm^−1^ and 2898 cm^−1^, respectively. All of these characteristic peaks confirm the stable chemical structure and purity of bLf [45,46]. After loading of bLf onto the surface of mineralization-encapsulated *L. rhamnosus* GG, the intensity of the amide I and amide II peaks decreased, indicating the successful entrapment of bLf [47]. The intensity of C–H stretching at 2898 cm^−1^ and 2937 cm^−1^ was also decreased, indicating the weakening of hydrogen bonding between the water molecules and C–H groups of alkyl chains. Interestingly, there was no shift in the peaks of bLf after loading onto the cells, indicating no chemical change in the composition of the mineralized gel on the cell surface. These results are consistent with previous reports [45,46].

**Figure 7:**
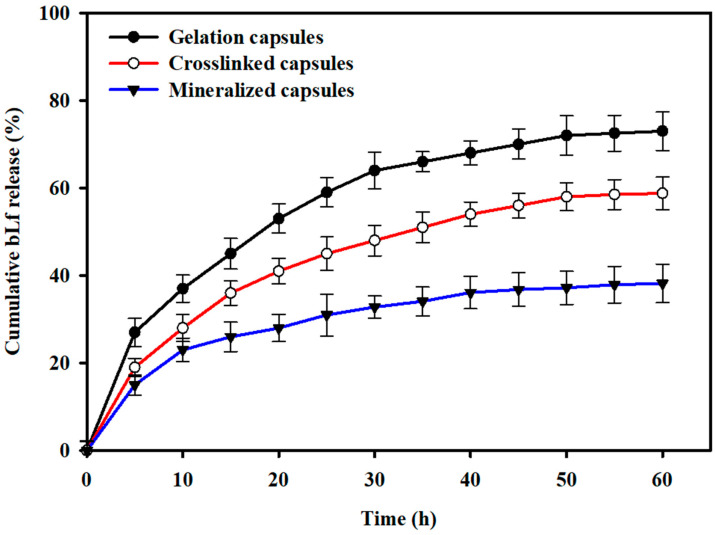
In vitro drug release profiles: The drug release from the gelation-encapsulated, crosslinking-encapsulated, and mineralization-encapsulated *L. rhamnosus* GG cells was studied in PBS at pH 7.4.

The in vitro release profile of bLf from the gelation-encapsulated, crosslinking-encapsulated, and mineralization-encapsulated *L. rhamnosus* GG cells is shown in Figure 7. The results show that the gelation-encapsulated, crosslinking-encapsulated, and mineralization-encapsulated *L. rhamnosus* GG cells presented a burst release during the initial 9 h and released approximately 36.2%, 28.7%, and 21.4% of the total loaded drug content, respectively. After the initial burst release, a sustained drug release was observed that leveled off after 60 h and showed cumulative drug release of 74.2%, 58.7%, and 37.5% for the gelation-encapsulated, crosslinking-encapsulated, and mineralization-encapsulated *L. rhamnosus* GG cells, respectively.

## 4. Discussion

As a novel approach, the microencapsulated probiotics (i.e., *L. rhamnosus* GG) were used as the carriers for biotherapeutics (i.e., bLf). Microencapsulation of *L. rhamnosus* GG was achieved through the liquid-droplet-forming method by using different polyelectrolytes.

For studying the swelling behavior and cell viability and morphology, three types of capsules—i.e., G-capsules, C-capsules, and M-capsules—of different sizes were prepared. The formation of G-capsules involved a simple gelation process, where the Ca^2+^ ions (i.e., cations) diffused into the alginate chains (i.e., anion) and interacted with the free functional groups in the polymer skeleton. This diffusion of Ca^2+^ ions into the alginate skeleton is highly favorable because of the small size of the former. During the formation of capsules, Tween 20 and xanthan gum favor the formation of tailless spherical capsules [48]. Both the shape and size of capsules are important determinants of the efficacy of the encapsulated system. For instance, although the large capsules allow more space for the encapsulated materials—such as cells, enzymes, drugs, etc. [49]—these are vulnerable to shear stress. The addition of capsules to the CaCl_2_ solution favored the thickening of the capsules and formed the C-capsules. Finally, M-capsules were formed through the precipitation of Na_2_HPO_4_ on the C-capsules. The modification of the cell surface by using different polymers and polyelectrolytes can be achieved either through hydrogel coating or the biomimetic mineralization method, as reported in our previous study [26]. In general, this process involves the electrostatic interaction between two oppositely charged polyelectrolytes. In our previous study, the oppositely charged poly(acrylate sodium) (PAS) and poly(diallyldimethylammonium chloride) (PDADMAC) formed a stable hydrogel on the cell surface. Due to the electrostatic interaction between PAS (positively charged) and PDADMAC (negatively charged), a complex with narrow size distribution was formed, which was colloidally stable and dispersible in water. This approach was used for the surface modification of *Saccharomyces cerevisiae* [26]. In another study, a mineralized structure composed of the positively charged Ca^2+^ and the negatively charged PO_4_^3−^, with an additional layer of magnetic nanoparticles, was formed on the surface of *Escherichia coli*. The modified cells efficiently responded to magnetic stimulus and moved under the influence of an external magnetic field [12].

It is well-established that the surface morphology of the capsule determines its physiological behavior and, thus, contributes to the performance of the encapsulated systems. For instance, a porous surface allows the diffusion of small molecules such as nutrients, gases, metabolites, etc. [50]. Herein, the more compact M-capsules (as opposed to the C-capsules and G-capsules) are likely to be more stable in the physiological environment. The swelling of all types of capsules in the order of M-capsules > C-capsules > G-capsules until a plateau point in SIF indicates their stability in SIF, which is a favorable characteristic for retaining probiotic bacteria. Furthermore, the monovalent ions present in the gastric juice in the stomach compete with the carboxylic groups of the gel and may cause the dissolution of capsules for the release of bacteria [18].

The effectiveness of probiotic encapsulation or surface modification is mainly evaluated by determining their viability under harsh environmental conditions, such as the pH of the GIT during oral delivery. The encapsulation or surface modification of microbial and non-microbial cells via different techniques requires that both the modification process and the materials used for surface modification should not affect their growth in any way. Furthermore, the entrapped drug in the encapsulation layer—bLf in this study—should not be toxic to the bacterial cells. Therefore, the viability of encapsulated *L. rhamnosus* GG cells was determined from three perspectives, i.e., the effect of encapsulation itself on the growth of bacteria, the effect of the modifying materials on the growth, and the effect of the toxicity of the entrapped drug on the cell viability. The encapsulated *L. rhamnosus* GG cells largely remained viable for an extended time period (Figure 5A), indicating that the microencapsulation did not significantly affect their growth. Similarly, the viability of encapsulated probiotics and the effectiveness of encapsulation or surface modification processes largely depend on the materials used. The encapsulation materials should be not only non-bactericidal, but also biocompatible and nontoxic towards different cells and tissues in the GIT. The relatively high growth of mineralization-encapsulated *L. rhamnosus GG* compared to the gelation-encapsulated and crosslinked-encapsulated cells indicates that encapsulation is protective in nature to preserve the viability of the cells. These results show that the materials used (i.e., alginate, calcium chloride, and disodium hydrogen phosphate) for the microencapsulation of *L. rhamnosus* GG cells are not only nontoxic, but also provide protection to the cells against harsh environmental conditions. In general, polycations in synthetic polyelectrolytes are toxic and kill cells by penetrating the cellular membrane [51]. In contrast, polycations composed of natural polymers such as alginate [52], cellulose [53], polylysine [54], polypeptides [55], hyaluronic acid [56], etc., are nontoxic and, thus, could be used for the encapsulation of cells. It has been reported that the use of alginate as a coating material helps to increase the mechanical strength and resist the chelating activity of Ca^2+^ ions [57]. The formation of colonies on the MRS agar plate by the microencapsulated *L. rhamnosus* GG after the release of bLf indicates the nontoxic effect of the drug towards the probiotic. This observation shows that the encapsulated *L. rhamnosus* GG cells could be used as carriers for other beneficial biotherapeutics without interfering with cell viability. Overall, these results demonstrate that microencapsulation is an important phenomenon to provide protection to probiotics against harsh environmental conditions such as varying pH, temperature, ionic strength, and others, and is a suitable approach for the delivery of biotherapeutics for treating different diseases.

Microencapsulation of biotherapeutic drugs in biocompatible polymers could be a promising approach for enhanced and controlled delivery. For instance, developing capsule structures through electrostatic interactions could be a preferred choice, as it allows a direct modification of the protein-based biotherapeutic or the use of harsh solvents, which may otherwise decrease the activity of the drug [58]. An effective coating or encapsulating material should possess the desired physicochemical properties to allow the absorption of drugs and favor their controlled release under different physiological environments. To date, the potential of various natural polymers (such as alginate [59], cellulose [60,61], chitosan [62], carrageenan [63], etc.) and synthetic polymers (such as [64]) to form complexes with different drugs (such as bovine serum albumin, gelatin, thrombin, soy protein, casein, etc.) has been evaluated, indicating their suitability to serve as carriers for the delivery of biotherapeutics. The findings of this study show that the amount of bLf entrapped on the surface of encapsulated *L. rhamnosus* GG cells increased with successive modification steps, which could be attributed to the increased surface porosity and availability of more functional groups on the modified cell surfaces, as evidenced from the FE-SEM micrographs (Figure 4). The comparatively high release of bLf from the gelation-encapsulated and crosslinking-encapsulated cells could be attributed to the low stability of the chelating structure in PBS. Furthermore, this high drug release could also be attributed to the large pore size due to the crosslinking of the alginate layer with the calcium chloride. Usually, the proteins entrapped in a gelation matrix are released by two mechanisms: diffusion of proteins through the porous matrix, and through degradation of the polymer network [65]. Due to the highly porous structure and high surface area of the encapsulated cells, the release medium diffuses into the interior of the layers and allows the dissolved bLf to diffuse into the medium. Thus, rapid diffusion of bLf from the gelation-encapsulated and crosslinking-encapsulated *L. rhamnosus* GG cells was achieved. In fact, bLf was loaded on the surface-modified cells through ionic bonding, both on the surface and within the gelation matrix, as indicated by the FTIR spectrum of mineralization-encapsulated *L. rhamnosus* GG cells; thus, high content of bLf was retained in the matrix. The strong ionic interactions prevented the burst release, and the surface-modified cells showed a sustained release. This sustained release from the mineralization-encapsulated cells could also be attributed to the stabilization of the disodium hydrogen phosphate by the phosphate ions from PBS [65]. These results show that the microencapsulated *L. rhamnosus* GG cells could serve as a promising probiotic drug delivery system.

The findings of this study show that the microencapsulated *L. rhamnosus* GG cells maintained their growth in SIF and SGF over time, confirming that the materials used for encapsulation were not only nontoxic but also provided protection to the encapsulated probiotics at the different pH levels of the GIT. The microencapsulated *L. rhamnosus* GG allowed the entrapment of bLf and showed a sustained drug release over time with no toxicity towards the probiotics. Thus, it can be concluded that microencapsulated probiotics could be an ideal platform for the oral delivery of biotherapeutics without compromising their viability for use in treating various diseases.

## 5. Conclusions

Herein, we reported the development of a novel approach to using microencapsulated probiotics (i.e., *L. rhamnosus* GG) as the carrier for biotherapeutics (i.e., bLf). The drug (i.e., bLf) and the selected polyelectrolytes (i.e., sodium alginate, calcium chloride, and disodium hydrogen phosphate) did not show any toxicity to the encapsulated *L. rhamnosus* GG cells, and the latter provided protection to the encapsulated probiotics at varying pH levels (i.e., SGF and SIF). Furthermore, the entrapped bLf showed a sustained drug release over time. FTIR spectroscopy confirmed the chemical interaction between the encapsulating materials and the probiotics, along with the successful entrapment of the drug (i.e., bLf). The varying surface morphology, size, and porosity of different capsules (i.e., G-capsules, C-capsules, and M-capsules) favored varying swelling behavior and drug-loading efficiency. All of these findings indicate the suitability of the system for simultaneous delivery of probiotics and drugs and, thus, could be an ideal platform for the oral delivery of biotherapeutics without compromising the viability of probiotics for use in treating various diseases.

## Figures and Tables

**Table 1:** The entrapment efficiency of bLf entrapped on the differently encapsulated *L. rhamnosus* GG cells.

Samples	Capsule Size (mm)		Entrapment Efficiency (%)
	Before Drug Loading	After Drug Loading	
bLf-entrapped gelation-encapsulated cells	3.20 ± 0.22	3.41 ± 0.22	78 ± 2.25
bLf-entrapped crosslinking-encapsulated cells	3.25 ± 0.31	3.43 ± 0.18	88 ± 1.78
bLf-entrapped mineralization-encapsulated cells	3.38 ± 0.35	3.61 ± 0.45	95 ± 1.12

## Data Availability

Data sharing not applicable.
